# A Systematic Review of Factors Associated with Mortality among Patients with *Mycobacterium avium* Complex Lung Disease

**DOI:** 10.3390/pathogens12111331

**Published:** 2023-11-08

**Authors:** Nobuhiro Fujishima, Kosaku Komiya, Mari Yamasue, Kazufumi Hiramatsu, Jun-ichi Kadota

**Affiliations:** 1 Respiratory Medicine and Infectious Diseases, Faculty of Medicine, Oita University, 1-1 Idaigaoka, Hasama-machi, Yufu 879-5593, Oita, Japansai-mari@oita-u.ac.jp (M.Y.);; 2 Medical Safety Management, Faculty of Medicine, Oita University, 1-1 Idaigaoka, Hasama-machi, Yufu 879-5593, Oita, Japan

**Keywords:** nontuberculous mycobacterium, *Mycobacterium avium* complex, prognosis, predictor, mortality

## Abstract

Purpose: As the number of patients with *Mycobacterium avium* complex lung disease is significantly increasing worldwide, several studies have focused on the prognostic factors associated with the disease. This systematic review investigated the factors associated with mortality among patients with *Mycobacterium avium* complex lung disease. Methods: Two investigators independently identified studies that were designed to determine risk factors for mortality in patients with *Mycobacterium avium* complex lung disease from PubMed, the Cochrane Register of Control Trial database, and EMBASE (accessed on 25 November 2022). Results: Of the 1133 titles and abstracts screened, 54 full texts were selected for review, and 15 studies were finally included in this systematic review. The most commonly studied risk factors were advanced age and low body mass index (11 studies for each), followed by male sex (8 studies), hypoalbuminemia (5 studies), and cavity (5 studies). In each study, these factors were mostly associated with increased all-cause mortality among patients with *Mycobacterium avium* complex lung disease as confirmed via multivariate analysis. Conclusions: Advanced age, male sex, low body mass index, hypoalbuminemia, and cavity are likely to be the common risk factors for all-cause mortality among patients with *Mycobacterium avium* complex lung disease, suggesting that patients with these factors need to be carefully monitored.

## 1. Introduction

Nontuberculous mycobacteria (NTM) are ubiquitous environmental organisms, and the number of patients with NTM lung disease is significantly increasing worldwide [1]. *Mycobacterium avium* complex (MAC) is the most commonly isolated pathogen that is associated with NTM lung disease, accounting for more than 90% [2]. It is known that the disease progression rate of MAC lung disease is widely varied. A large cohort study focusing on the natural history of MAC lung disease reported that approximately 60% of the patients showed a progressive clinical course resulting in treatment initiation within 3 years of diagnosis, whereas around 25% exhibited stable MAC lung disease for at least 3 years [3]. However, it should be noted that the efficacy of treatment for this disease even when multidrug regimens are used has not reached satisfactory levels. The treatment success rate of multidrug regimens containing macrolides was reported to be approximately 60% [4]. Consequently, it has not yet been determined when the treatment for MAC lung diseases should be administered.

A recent systematic review regarding the prognosis of patients with MAC lung disease indicated a five-year all-cause mortality of approximately 27% (95% CI 21.3–37.8%) [5], which seems to be coordinative to other poor prognostic lung diseases, including lung cancer and interstitial pneumonia [6]. To effectively manage patients with MAC lung disease, timely treatment initiation should be based on the prognostic factors [7]. While a number of studies have investigated the prognostic factors of MAC lung disease, no systematic review regarding these interests has been conducted. Therefore, we systematically reviewed published articles that are designed to determine the factors associated with mortality among patients with MAC lung disease. This study aims to summarize the prognostic factors of MAC lung disease to provide important information for decision-making about the treatment strategy in clinical practice.

## 2. Methods

### 2.1. Search Strategy

This systematic review was conducted in accordance with the guidelines of the Preferred Reporting Items for Systematic Reviews and Meta-analyses (PRISMA) statement (Supplementary File S1). To ensure timely publication, this review has not been registered in any database in advance. In this systematic review, studies that evaluated the factors associated with any types of mortality in patients with MAC lung disease which were diagnosed in accordance with the criteria of the American Thoracic Society (ATS), the Infectious Diseases Society of America (IDSA) 2007 or ATS, the European Respiratory Society (ERS), the European Society of Clinical Microbiology and Infectious Diseases (ESCMID), and IDSA 2020 guidelines were included [8,9]. Since the risk factors for mortality in patients with MAC lung disease may be confounded each other, we restricted the inclusion criteria to studies that performed statistical adjustments by multivariate analysis in order to exclude low quality studies.

We searched for the studies using PubMed and Cochrane CENTRAL Register of Controlled Trials (CENTRAL) database from 1 January 2007 to 31 July 2022. The combinations of the following search terms were applied: “nontuberculous mycobacterium” or “nontuberculosis mycobacterium” or “nontuberculous mycobacterium” or “nontuberculosis mycobacterium” or “mycobacterium avium complex” or “mycobacterium avium” or “m avium” or “mycobacterium intracellulare” or “m intracellulare” or “prognosis” or “mortality” (assessed on 25 November 2022). This systematic review included additional studies quoted by articles identified using the above search criteria in order to collect as many studies as possible.

Studies on the diagnostic methods of MAC lung diseases that were not conducted in accordance with the criteria by the guidelines 2007 or 2020 and those in which multivariate analysis was not conducted or statistical methods were not clearly stated were excluded. Publications written in languages other than English or in abstract form alone were not included. We also excluded studies targeting children, patients limited with specific severity (i.e., mild cases or severe cases only), or patients limited with specific backgrounds (i.e., cystic fibrosis or immunocompromised conditions). The title, abstracts, and full text articles were screened and further evaluated by two authors (NF and MY) independently. Disagreements were resolved by the decision of a third reviewer (KK).

### 2.2. Data Extraction and Analysis

We extracted the following information from the included studies: study design, nationality, sample size, enrolled age groups, treatment status, follow-up period, mortality rate, and the factors associated with all-cause or disease-specific mortality in patients with MAC lung disease in accordance with the multivariate analyzes. Regardless of the statistical significance of the results by multivariate analysis, the factors associated with mortality that were analyzed in two or more studies were documented. Moreover, the factors that were analyzed in a multivariate analysis in a single study (never assessed in other studies) were not included. Meta-analysis was not performed due to the variability in outcomes and its definitions.

### 2.3. Assessing the Risk of Bias

The risk of bias was assessed using the scales of the Risk of Bias Assessment tool for nonrandomized studies (RoBANS) [10]. The following six domains were assessed for the RoBANS: (1) selection of participants, (2) confounding variables, (3) measurements of exposure, (4) blinding of outcome assessments, (5) incomplete outcome data, and (6) selective outcome reporting. Each domain was assessed as having low, high, or unclear risk of bias. The assessments of the studies were conducted by two investigators (NF and MY) independently, and disagreements were resolved by a third reviewer (KK).

## 3. Results

**Figure 1:**
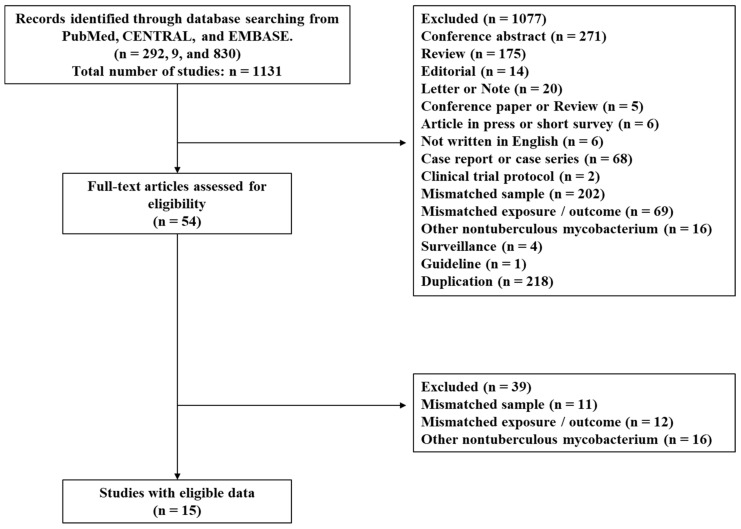
Flow diagram of study selection.

We identified 292, 9 and 830 studies from PubMed, the CENTRAL, and EMBASE, respectively, and 1077 studies were excluded as the title and abstract did not meet the inclusion criteria. Of the remaining 54 records, 39 were excluded after retrieving and inspecting the full text as shown in Figure 1. We eventually included 15 studies in this systematic review (13 retrospective and 2 prospective observational studies), in which 13 of the included studies have been published from Japan (Table 1). While 13 of the 15 studies focused on all-cause mortality as an outcome [11,12,13,14,15,16,17,18,19,20,21,22,23], one study excluded death due to malignancy [24] and another study did not clearly define whether death denotes all-cause mortality [25].

Among the 15 studies, multivariate analysis was performed on advanced age in 11 studies [11,12,14,15,16,17,18,19,20,21,23]; low body mass index (BMI) in 11 studies [11,12,13,14,15,16,18,19,21,23,24]; male sex in eight studies [11,15,16,18,19,21,23,24]; cavity [14,15,19,22,24] and hypoalbuminemia [11,17,20,21,23] in five studies; fibrocavitary (FC) pattern in four studies [11,17,21,23]; respiratory comorbidity [13,20,23], and *Aspergillus* co-infection [15,17,20] in 3 studies for each; and weakened erector spinae muscles (ESM) [18,19], low forced vital capacity % (FVC %) [12,19], diabetes mellitus [15,16], malignancy [16,21], nodular bronchiectatic (NB) pattern [14,19], bronchiectasis [14,22], pleuroparenchymal fibroelastosis (PPFE) [11,14], the presence of lung parenchymal consolidation [14,25], C-reactive protein (CRP) level [11,15], and sputum smear grade [11,16] in two studies for each. Reported significance associated with increased mortality was more frequently observed in advanced age, male sex, low BMI, hypoalbuminemia, decreased lung function, respiratory comorbidities including *Aspergillus* co-infection, cavity, FC pattern, PPFE, and CRP level, whereas no statistical significance was observed in decreased ESM, diabetes mellitus, malignancy, NB pattern, bronchiectasis, consolidation, and smear grade with a small number of studies in each variable (Table 2).

*Advanced age:* Advanced age was studied via multivariate analysis in 11 studies [11,12,14,15,16,17,18,19,20,21,23]. Six studies analyzed the patients’ age as a continuous variable [11,12,14,15,18,19], of which three found advanced age as a significant risk factor for all-cause mortality (range of HR: 1.04–1.08) [11,12,15], while three studies did not. The other five studies analyzed the patient’s age as a categorical variable, and all studies showed that old age groups were associated with increased all-cause mortality (range of HR: 1.919–3.672) [16,17,20,21,23]. However, the definition of old age varied according to the studies (≥65 [16] and ≥70 [17,20,21,23]).

*Male sex:* Male sex was studied via multivariate analysis in eight studies [11,15,16,18,19,21,23,24]. Six studies reported a significant association with mortality (range of HR: 1.839–4.62) [11,15,16,18,21,23], whereas two studies did not [19,24].

*Low BMI:* BMI was studied via multivariate analysis in 11 studies [11,12,13,14,15,16,18,19,21,23,24]. Seven studies used the data as a continuous variable [11,12,13,14,15,16,18], and five studies found a significant association between decreased BMI and all-cause mortality (range of HR: 0.61–0.858) [12,14,15,16,18]. Five studies (one study on both continuous and categorical variables [18]) analyzed BMI as a categorical variable with a common cutoff value (<18.5 kg/m^2^) [18,19,21,23,24], of which four reported that the low BMI groups were significantly related to mortality (range of HR: 1.881–4.62) [19,21,23,24], whereas one study did not find an association [13].

*Hypoalbuminemia:* Hypoalbuminemia was studied via multivariate analysis in five studies [11,17,20,21,23]. One study used the data as a continuous variable [11], and four studies defined hypoalbuminemia as a common cutoff value (serum albumin level ≤3.5 g/dL) [17,20,21,23], and a significant association with all-cause mortality was found in all these studies (range of HR: 2.497–4.78).

*ESM:* ESM was studied via multivariate analysis in two studies [18,19]. Asakura et al. found that the cross-sectional area of ESM, which was measured on chest CT, was not significantly associated with all-cause mortality when adjusted with the current treatment status and respiratory functions [19]. Akahori et al. analyzed the role of the mean attenuation of ESM along with the cross-sectional area of ESM [18], and the results indicated that the mean attenuation of CT value on ESM was significantly associated with worse survival (HR: 2.512, *p* = 0.012), whereas the cross-sectional area of ESM was not.

*Low predicted FVC %:* Predicted FVC % was studied via multivariate analysis in two studies [12,19], indicating a significant association with all-cause mortality (range of HR: 0.95–0.96) in both studies.

*Aspergillus*: *Aspergillus* co-infection was studied via multivariate analysis in three studies [15,17,20]. Shirai et al. reported that the positivity of *Aspergillus* precipitating antibody, which was tested within one month of MAC lung disease diagnosis, was a significant risk factor for all-cause mortality (HR, 2.729; 95% CI, 1.247–5.973) [17]. Furuuchi et al. showed that the isolation of *Aspergillus fumigatus* from respiratory specimens was significantly associated with all-cause mortality (HR, 3.546; 95% CI, 1.435–8.092) [20]. In contrast, Fukushima et al. reported that chronic pulmonary aspergillosis was not related to all-cause mortality (HR, 7.32; 95% CI, 0.998–35.55) [15].

*Respiratory comorbidity:* Respiratory comorbidity was studied via multivariate analysis in three studies [13,20,23], all of which found significant associations with all-cause mortality (range of HR: 1.454–3.01). However, the definition of respiratory comorbidity varied. Furuuchi et al. defined respiratory comorbidities as old pulmonary tuberculosis, pulmonary emphysema, interstitial pneumonia, and asthma [20]. Hayashi et al. included pulmonary tuberculosis, pulmonary emphysema, interstitial pneumonia, lung cancer, asthma, and pneumoconiosis [23]. Yagi et al. defined the comorbidities as old pulmonary tuberculosis, chronic obstructive pulmonary disease, interstitial lung disease, lung cancer, and chronic pulmonary aspergillosis [13].

*Diabetes mellitus:* Diabetes mellitus was studied via multivariate analysis in two studies [15,16], of which one found a significant relationship between diabetes mellitus and all-cause mortality (HR, 2.84; 95% CI, 1.20–6.40) [15], while the other study did not (HR, 1.348; 95% CI, 0.723–2.514) [16].

*Malignancy:* Malignancy was studied via multivariate analysis in two studies [16,21], of which one found a significant association between malignancy and all-cause mortality (HR, 1.98; 95% CI, 1.23–3.18) [21], while the other study did not [16].

*Cavity:* Lung cavity was studied via multivariate analysis in five studies [14,15,19,22,24], of which three analyzed the presence of cavity on chest CT as a categorical variable [15,19,24], whereas two analyzed it as a scored continuous variable using chest CT [14] or chest X-ray [22]. As a result, the significant association between cavity and mortality was observed in four studies (range of HR, 1.326–5.84) [15,19,22,24].

*FC pattern:* FC pattern was studied via multivariate analysis in four studies [11,17,21,23], all of which reported that FC disease was a significant factor related to all-cause mortality (range of HR, 1.695–3.063). FC pattern was determined on the basis of chest CT features in these studies.

*NB pattern:* NB pattern was studied via multivariate analysis in two studies [14,19], and both reported that NB pattern was not a significant factor related to all-cause mortality.

*Bronchiectasis:* Bronchiectasis was studied via multivariate analysis in two studies [14,22], of which one scored pulmonary bronchiectasis on chest X-ray and found a significant relationship between high score and all-cause mortality [14], whereas the other study scored pulmonary bronchiectasis on chest CT and did not find a significant association with all-cause mortality [22].

*PPFE:* PPFE feature was studied via multivariate analysis in two studies [11,14], both of which reported that the feature was a significant factor related to all-cause mortality (range of HR, 1.66–4.78). PPFE was determined on the basis of chest CT features in these studies.

*Consolidation:* Pulmonary parenchymal consolidation was studied via multivariate analysis in two studies [14,25]. Wang et al. scored pulmonary consolidation on chest CT and found a significant relationship between high score and mortality (HR, 6.0; 2.3–15.5) in multivariate analysis [25]. In contrast, the other study evaluated the consolidation in each lobe on chest X-ray using a scoring system and did not find a significant association with all-cause mortality [14].

*CRP:* Serum CRP level was studied via multivariate analysis in two studies [11,15]. While one study reported that CRP level was a significant factor related to all-cause mortality (HR, 1.22) [15], the other study did not find the association [11].

*Smear grade:* Acid-fast bacilli smear grade was studied via multivariate analysis in two studies [11,16], of which one study reported that the results varied by smear grade [16], whereas the other study did not find a significant association with all-cause mortality [11].

### Assessing the Risk of Bias

In the scale for the RoBANS, the average number for “high” risk of bias across the six domains was approximately 2.2 for 14 studies assessing the prognostic factors for MAC lung disease (Table 3). The quality of studies regarding “measurement of exposure” and “blinding of outcome assessments” was good, whereas the quality concerning the “selection of participants”, “confounding variables”, and “incomplete outcome data” was poor. The main reasons for the low quality concerning these domains were that almost of all studies were uncontrolled retrospective cohort studies. The domain of “selective outcome reporting” was unclear in all studies for the reason that no available experimental protocol of these studies and the pre-defined primary/secondary outcomes were not described as planned.

## 4. Discussion

This systematic review determined 15 factors that were analyzed as potential predictors for mortality. These were classified into the patient’s backgrounds in two factors (advanced age and male sex), nutritional status and respiratory function in four factors (low BMI, hypoalbuminemia, predicted FVC%, and ESM), comorbidities in four factors (aspergillus co-infection, respiratory comorbidity, diabetes mellitus, and malignancy), chest radiological findings in six factors (cavity, FC pattern, NB pattern, bronchiectasis, PPFE, and consolidation), and laboratory data in two factors (CRP level and smear grade). Among them, the reported significance associated with increased mortality was more frequently observed in advanced age, male sex, low BMI, hypoalbuminemia, decreased lung function, respiratory comorbidities including *Aspergillus* co-infection, cavity, and FC pattern.

Both advanced age and male sex were reported to be poor prognostic factors in a variety of respiratory diseases [26,27,28]. Differences in smoking habits, dust exposures, and socioeconomic status between males and females were considered as a mechanism to explain the reason. The role of sex hormones in the regulation of the immune system may also contribute to the sex differences in the severity of infectious and noninfectious respiratory diseases [29]. The result indicating that male sex was associated with all-cause mortality in patients with MAC lung diseases was consistent with those obtained from already-published literatures.

BMI and serum albumin level are generally used as markers of nutritional status [30]. Low BMI and hypoalbuminemia are known to be associated with increased mortality in patients with not only acute infectious diseases [31,32] but also chronic pulmonary diseases including obstructive pulmonary diseases (COPD) and lung cancer [33,34]. Decreased BMI may deteriorate lung function which is another potential risk factor for poor prognosis. Furthermore, it is known that albumin itself has an anti-inflammatory effect leading to the attenuation of tissue damage inhibiting TNF-α and oxidant production [35,36]. BMI, serum albumin, and lung function may be confounded with each other, and this complex may play a major role in the prognosis of chronic respiratory diseases, including MAC lung diseases. No significant tendency of which factor predominantly contributes to the prognosis has not been observed through this systematic review.

ESMs are located at the back, which allow various movements from stretching to aerobic back-bends, and have been reported to be associated with the severity and prognosis of COPD [37], idiopathic pulmonary fibrosis [38], and community-acquired pneumonia among the elderly [39]. ESMs are the primary muscles involved in breathing [40] and may be correlated with BMI, nutritional status, and lung function [41,42]. However, the present results regarding the impact of ESM on mortality varied [18,19], which could be due to the differences in measuring methods among the investigators. Due to the limited number of the studies, no conclusive evidence regarding the impact of ESM on the prognosis of MAC lung disease was obtained.

Pulmonary comorbidities are generally considered as poor prognostic factors in any types of pulmonary diseases [43,44]. Although the definitions of pulmonary comorbidities in the included studies varied [13,20,23], emphysema and interstitial pneumonia were commonly included. These diseases influence the overall survival and may increase the risk of pulmonary infections. For example, obstructive or restrictive ventilatory impairment might make the drainage of pathogens from the airway ineffective. In fact, the antibiotic treatment period would be prolonged to complete the cure of bacterial pneumonia especially for COPD patients infected with *Pseudomonas aeruginosa* [45]. Furthermore, these lung abnormalities lead to a susceptibility to *Aspergillus* infection, which may accelerate the tissue destruction through inflammatory responses [46]. Azole antifungal drugs are used as the first treatment of choice for pulmonary *Aspergillus* diseases [47]. However, treatment with azoles necessitates the avoidance of rifampicin, which is a major drug against MAC lung disease, due to drug interactions [48]. This fact complexifies the treatment strategy for patients with MAC lung diseases complicated with *Aspergillus* infection, leading to a poor management of the diseases.

Cavitary lesion and FC pattern were indicated as potential risk factors for mortality in the present systematic review. Moreover, these features reflect advanced MAC lung disease, and the presence of cavity itself can reduce the efficacy of chemotherapy [49]. Possible mechanisms include the rapid bacterial growth in the anaerobic environment of the cavity, reduced reach of the drug to the luminal surface, and spread of the bacteria from the cavity to other areas of the lungs [50].

The strength of this systematic review is that it is the first study to describe the factors associated with the mortality of MAC lung disease targeting general patient samples. We found that advanced age, male sex, low BMI, hypoalbuminemia, decreased lung function, respiratory comorbidities including *Aspergillus* co-infection, cavity, and FC pattern were likely to be common risk factors for all-cause mortality among patients with MAC lung disease. However, it should be determined how these results can be incorporated in clinical practice. If the associated factor exacerbates the disease progression with one direction (i.e., malignancy or diabetes mellitus), the intervention to control the comorbidities would improve the disease prognosis in MAC lung disease. If the factor exacerbates the disease progression and vice versa (i.e., poor nutritional status or low lung function), interrupting the vicious circle might improve the prognosis. Unfortunately, no solid evidence of the interventional efficacy has been established to address these questions. Furthermore, it has not yet been determined whether prompt chemotherapy initiation should be recommended for patients with these poor prognostic factors. The official ATS/ERS/ESCMID/IDSA 2020 guidelines recommended the initiation of treatment rather than watchful waiting, especially in the context of cavitary lung disease [9]. Nevertheless, an interventional study has never been conducted to clarify the efficacy of early chemotherapy initiation. Indeed, no study that assessed the timing of chemotherapy initiation was found in this systematic review. To clarify when chemotherapy should be start, we need a randomized control study that adjusts for the patients’ baseline severity based on the prognostic factors obtained from this systematic review.

However, this systematic review has several limitations. First, as shown in the risk of bias assessment, patient selection bias existed in each study. Of the 15 studies, 13 were published in Japan, and this heterogeneity could interfere with the generalizability. More studies conducted in other countries are needed. Second, the cutoff values for contentious variables varied especially in the categorization of “advanced age”. A common definition or cutoff value is needed to evaluate the impact of each factor more objectively. Third, focused factors and adjustment factors differed in each study. Some studies evaluated the radiological features with the patients’ background and nutritional status, but other studies did not consider. This fact may lead to evaluation bias, even when we included the factors that resulted from multivariate analyzes in each study. Finally, we could not conduct a meta-analysis in this systematic review because the definitions of each variable differed among the included studies and majority of the variables may be confounded with each other.

## 5. Conclusions

In conclusion, this systematic review showed that advanced age, male sex, low BMI, hypoalbuminemia, decreased lung function, respiratory comorbidities including *Aspergillus* co-infection, cavity, FC pattern, and PPFE were likely to be common risk factors for all-cause mortality among patients with MAC lung disease. Physicians need to be aware of these associated factors and should carefully monitor high-risk patients. However, no clear evidence can confirm whether intervention to control these risk factors or early chemotherapy for these patients should be indicated. Randomized control studies are required to determine the efficacy of the interventions, along with a large-scaled cohort to confirm the risk factors raised in this systematic review.

## Figures and Tables

**Table 1:** Characteristics of the studies included in this systematic review.

Author, Publication Year	Study Design, Nationality	Number of Samples	Patients’ Age	Number of Patients Treated (%) with Chemotherapy, Regimens	Follow-Up Period (Year)	Number of Death (%)
**Aono 2022 [11]**	Retrospective, Japan	97 with and 721 without PPFE	74.4 ± 9.9 with and 68.7 ± 10.9 without PPFE (mean ± SD)	62 (64) with and 466 (65) without PPFE	3.1, 1.4–5.1 with and 4.5, 2.0–6.9 without PPFE (median, IQR)	93 (11)
**Takasaka, 2022 [24]**	Retrospective, Japan	174	73 ± 9.8 (mean ± SD)	68 (39), RECAM *	3.4, 2.2–5.1 (median, IQR)	20 (12)
**Ogawa, 2021 [12]**	Prospective, Japan	269	68, 62–75 (median, IQR)	142 (53), n.d.	4.1	22 (8)
**Yagi K, 2021 [13]**	Retrospective, Japan	54 with and 54 without pleuritis	73, 67–81 with and 77, 70–82 without pleuritis (median, IQR)	60 (56), various	0.8, 0.2–2.3 with and 4.9, 3.3–5.7 without pleuritis (median, IQR)	33 (31)
**Yamamoto, 2021 [14]**	Retrospective, Japan	224	68, 62–74 (median, IQR)	86 (38), RECAM *	n.d.	9 (4)
**Fukushima, 2021 [15]**	Retrospective, Japan	295	65.9 ± 10.5 (mean ± SD)	295 (100), various	n.d.	48 (16)
**Wang, 2020 [25]**	Retrospective, Taiwan	123	66.7 ± 14.2 (mean ± SD)	47 (38), various	4.15 ± 2.52 (mean ± SD)	22 (18)
**Moon, 2020 [16]**	Retrospective, South Korea	663	64.1 ± 11.8 (mean ± SD)	306 (46), various	3.9 ± 2.8 (mean ± SD)	63 (10)
**Shirai, 2020 [17]**	Retrospective, Japan	131	72, 63–79 (median, IQR)	95 (73), RECAM *	4.0, 2.2–5.7 (median, IQR)	34 (26)
**Akahori, 2019 [18]**	Retrospective, Japan	248 (137 and 111 in different cohorts)	73, 65–78 and 76, 67–82 (median, IQR)	85 (66) and 58 (52), RECAM	4.6, 3.3–6.9 and 5.8, 2.7–5.8 (median, IQR)	44 (18)
**Asakura, 2018 [19]**	Prospective, Japan	260	69, 64–76 (median, IQR)	110 (42), n.d.	3.0, 2.0–3.8 (median, IQR)	21 (8)
**Furuuchi, 2018 [20]**	Retrospective, Japan	329 (40 with and 289 without *Aspergillus* co-infection)	74, 67–79 with and 73, 65–78 without *Aspergillus* co-infection (median, IQR)	177 (54), n.d.	3.7, 2.0–5.1 (median, IQR)	67 (20)
**Kumagai, 2017 [21]**	Retrospective, Japan	368	72 ± 10 (mean ± SD)	169 (46), various	3.5, 0–9.3 (median, IQR)	75 (21)
**Furuuchi, 2017 [22]**	Retrospective, Japan	218	71 ± 9.6 (mean ± SD)	114 (52), various	3.3, 0–5.5 (median, IQR)	32 (15)
**Hayashi, 2012 [23]**	Retrospective, Japan	634	68.9 ± 11.4 (mean ± SD)	196 (31), various	4.7, 0–11.1 (median, IQR)	160 (25)

IQR, interquartile range; n.d., not described; PPFE, pleuroparenchymal fibroelastosis; RECAM, rifampicin; + ethambutol + clarithromycin; SD, standard deviation. * The number of patients treated with other regimens unknown.

**Table 2:** Summary of the significant or non-significant factors associated with mortality in patients with *Mycobacterium avium* complex lung disease.

	Significant	Non-Significant
**Patient’s backgrounds**		
**Advanced age**	[11] [12] [15] [16] [17] [20] [21] [23]	[14] [18] [19]
**Male sex**	[11] [15] [16] [18] [21] [23]	[19] [24]
**Nutritional status and lung function**		
**Low body mass index**	[12] [14] [15] [16] [18] ^a^ [19] ^b^ [21] [23] [24]	[11] [13] [18] ^a^ [19] ^b^
**Hypoalbuminemia**	[11] [17] [20] [21] [23]	
**Decreased erector spinae muscles**	[18] ^c^	[18] ^c^ [19] ^d^
**Low predicted forced vital capacity %**	[12] [19]	
**Comorbidity**		
***Aspergillus* ** **co-infection**	[17] [20]	[15]
**Respiratory comorbidity**	[13] [20] [23]	
**Diabetes mellitus**	[15]	[16]
**Malignancy**	[21]	[16]
**Chest radiological features**		
**Cavity**	[15] [19] [22] [24]	[14]
**Fibrocavitary pattern**	[11] [17] [21] [23]	
**Nodular bronchiectatic pattern**		[14] [19]
**Bronchiectasis**	[22]	[14]
**Pleuroparenchymal fibroelastosis**	[11] [14]	
**Consolidation**	[25]	[14]
**Laboratory data**		
**C-reactive protein**	[15]	[11]
**Smear grade**	[11] [16] ^e^	[16] ^e^

^a^ Results varied by contentious or categorical variables. ^b^ Results varied by analytical models in the same sample. ^c^ Significance found in the mean erector spinae muscle attenuation but not in the area of the erector spinae muscles on CT. ^d^ Results varied by analytical models in the same sample. ^e^ Results varied by analytical models in the same sample.

**Table 3:** Quality of studies included in this systematic review.

Studies	Selection of Participants	Confounding Variables	Measurements of Exposure	Blinding of Outcome Assessments	Incomplete Outcome Data	Selective Outcome Reporting
**Aono, 2023 [11]**	high	high	low	low	low	unclear
**Takasaka, 2022 [24]**	high	high	low	low	low	unclear
**Ogawa, 2021 [12]**	low	high	low	unclear	high	unclear
**Yagi K, 2021 [13]**	high	low	low	low	low	unclear
**Yamamoto, 2021 [14]**	high	high	low	high	high	unclear
**Fukushima, 2021 [15]**	high	high	low	low	high	unclear
**Wang, 2020 [25]**	high	high	low	low	low	unclear
**Moon, 2020 [16]**	high	high	low	low	high	unclear
**Shirai, 2020 [17]**	high	high	low	low	high	unclear
**Akahori, 2019 [18]**	high	high	low	low	high	unclear
**Asakura, 2018 [19]**	low	low	low	unclear	high	unclear
**Furuuchi, 2018 [20]**	high	high	low	low	low	unclear
**Kumagai, 2017 [21]**	high	high	low	unclear	low	unclear
**Furuuchi, 2017 [22]**	high	high	low	low	low	unclear
**Hayashi, 2012 [23]**	high	high	low	unclear	unclear	unclear

## Data Availability

Data sharing is not applicable due to systematic review article, but the article lists are available from the corresponding author on reasonable request.

## Supplementary Materials

The following supporting information can be downloaded at: https://www.mdpi.com/article/10.3390/pathogens12111331/s1, Supplementary File S1: PRISMA Checklist [51].

## References

[1] Namkoong H., Kurashima A., Morimoto K., Hoshino Y., Hasegawa N., Ato M., Mitarai S. (2016). Epidemiology of Pulmonary Nontuberculous Mycobacterial Disease, Japan. Emerg. Infect. Dis..

[2] Jhun B.W., Moon S.M., Jeon K., Kwon O.J., Yoo H., Carriere K.C., Huh H.J., Lee N.Y., Shin S.J., Daley C.L. (2020). Prognostic factors associated with long-term mortality in 1445 patients with nontuberculous mycobacterial pulmonary disease: A 15-year follow-up study. Eur. Respir. J..

[3] Hwang J.A., Kim S., Jo K.W., Shim T.S. (2017). Natural history of Mycobacterium avium complex lung disease in untreated patients with stable course. Eur. Respir. J..

[4] Diel R., Nienhaus A., Ringshausen F.C., Richter E., Welte T., Rabe K.F., Loddenkemper R. (2018). Microbiologic Outcome of Interventions Against Mycobacterium avium Complex Pulmonary Disease: A Systematic Review. Chest.

[5] Diel R., Lipman M., Hoefsloot W. (2018). High mortality in patients with Mycobacterium avium complex lung disease: A systematic review. BMC Infect. Dis..

[6] Wong A.W., Ryerson C.J., Guler S.A. (2020). Progression of fibrosing interstitial lung disease. Respir. Res..

[7] Kurz S.G., Zha B.S., Herman D.D., Holt M.R., Daley C.L., Ruminjo J.K., Thomson C.C. (2020). Summary for Clinicians: 2020 Clinical Practice Guideline Summary for the Treatment of Nontuberculous Mycobacterial Pulmonary Disease. Ann. Am. Thorac. Soc..

[8] Griffith D.E., Aksamit T., Brown-Elliott B.A., Catanzaro A., Daley C., Gordin F., Holland S.M., Horsburgh R., Huitt G., Iademarco M.F. (2007). An official ATS/IDSA statement: Diagnosis, treatment, and prevention of nontuberculous mycobacterial diseases. Am. J. Respir. Crit. Care Med..

[9] Daley C.L., Iaccarino J.M., Lange C., Cambau E., Wallace R.J., Andrejak C., Böttger E.C., Brozek J., Griffith D.E., Guglielmetti L. (2020). Treatment of Nontuberculous Mycobacterial Pulmonary Disease: An Official ATS/ERS/ESCMID/IDSA Clinical Practice Guideline. Clin. Infect. Dis..

[10] Kim S.Y., Park J.E., Lee Y.J., Seo H.-J., Sheen S.-S., Hahn S., Jang B.-H., Son H.-J. (2013). Testing a tool for assessing the risk of bias for nonrandomized studies showed moderate reliability and promising validity. J. Clin. Epidemiol..

[11] Aono Y., Hozumi H., Kono M., Hashimoto D., Nakamura H., Yokomura K., Imokawa S., Shirai M., Akahori D., Inoue Y. (2022). Prognostic significance of radiological pleuroparenchymal fibroelastosis in Mycobacterium avium complex lung disease: A multicentre retrospective cohort study. Thorax.

[12] Ogawa T., Asakura T., Suzuki S., Okamori S., Kusumoto T., Sato Y., Namkoong H., Kamata H., Ishii M., Fukunaga K. (2021). Longitudinal validity and prognostic significance of the St George’s Respiratory Questionnaire in Mycobacterium avium complex pulmonary disease. Respir. Med..

[13] Yagi K., Ito A., Fujiwara K., Morino E., Hase I., Nakano Y., Asakura T., Furuuchi K., Morita A., Asami T. (2021). Clinical Features and Prognosis of Nontuberculous Mycobacterial Pleuritis: A Multicenter Retrospective Study. Ann. Am. Thorac. Soc..

[14] Yamamoto Y., Tsujino K., Kuge T., Okabe F., Kawasaki T., Matsuki T., Kagawa H., Miki M., Miki K., Mori M. (2021). Pleuroparenchymal fibroelastosis in Mycobacterium avium complex pulmonary disease: Clinical characteristics and prognostic impact. ERJ Open Res..

[15] Fukushima K., Kitada S., Komukai S., Kuge T., Matsuki T., Kagawa H., Tsujino K., Miki M., Miki K., Kida H. (2021). First line treatment selection modifies disease course and long-term clinical outcomes in Mycobacterium avium complex pulmonary disease. Sci. Rep..

[16] Moon S.W., Lee E.H., Choi J.S., Leem A.Y., Lee S.H., Lee S.H., Kim S.Y., Chung K.S., Jung J.Y., Park M.S. (2020). Impact of prognostic nutritional index on outcomes in patients with Mycobacterium avium complex pulmonary disease. PLoS ONE.

[17] Shirai T., Furuuchi K., Fujiwara K., Nakamoto K., Tanaka Y., Ishii H., Yoshiyama T., Yoshimori K., Takizawa H., Sasaki Y. (2020). Impact of Aspergillus precipitating antibody test results on clinical outcomes of patients with Mycobacterium avium complex lung disease. Respir. Med..

[18] Akahori D., Suzuki Y., Yokomura K., Shirai M., Yasui H., Hozumi H., Karayama M., Furuhashi K., Enomoto N., Fujisawa T. (2019). Body composition changes successfully classify prognosis in patients with mycobacterium avium complex lung disease. J. Infect..

[19] Asakura T., Yamada Y., Suzuki S., Namkoong H., Okamori S., Kusumoto T., Niijima Y., Ozaki A., Hashimoto M., Yagi K. (2018). Quantitative assessment of erector spinae muscles in patients with Mycobacterium avium complex lung disease. Respir. Med..

[20] Furuuchi K., Ito A., Hashimoto T., Kumagai S., Ishida T. (2018). Clinical significance of Aspergillus species isolated from respiratory specimens in patients with Mycobacterium avium complex lung disease. Eur. J. Clin. Microbiol. Infect. Dis. Off. Publ. Eur. Soc. Clin. Microbiol..

[21] Kumagai S., Ito A., Hashimoto T., Marumo S., Tokumasu H., Kotani A., Yamaki H., Shirata M., Furuuchi K., Fukui M. (2017). Development and validation of a prognostic scoring model for Mycobacterium avium complex lung disease: An observational cohort study. BMC Infect. Dis..

[22] Furuuchi K., Ito A., Hashimoto T., Kumagai S., Ishida T. (2017). Clinical significance of the radiological severity score in Mycobacterium avium complex lung disease patients. Int. J. Tuberc. Lung Dis..

[23] Hayashi M., Takayanagi N., Kanauchi T., Miyahara Y., Yanagisawa T., Sugita Y. (2012). Prognostic factors of 634 HIV-negative patients with Mycobacterium avium complex lung disease. Am. J. Respir. Crit. Care Med..

[24] Takasaka N., Hosaka Y., Fukuda T., Shinfuku K., Chida K., Shibata S., Kojima A., Hasegawa T., Yamada M., Yamanaka Y. (2022). Impact of emphysema on the prognosis of Mycobacterium avium complex pulmonary disease. Respir. Med..

[25] Wang P.-H., Pan S.-W., Shu C.-C., Chen C.-Y., Wei Y.-F., Cheng S.-L., Wang H.-C., Yu C.-J. (2020). Clinical course and risk factors of mortality in Mycobacterium avium complex lung disease without initial treatment. Respir. Med..

[26] Falagas M.E., Mourtzoukou E.G., Vardakas K.Z. (2007). Sex differences in the incidence and severity of respiratory tract infections. Respir. Med..

[27] Ley B., Ryerson C.J., Vittinghoff E., Ryu J., Tomassetti S., Lee J.S., Poletti V., Buccioli M., Elicker B.M., Jones K.D. (2012). A multidimensional index and staging system for idiopathic pulmonary fibrosis. Ann. Intern. Med..

[28] Marrie T.J. (2000). Community-acquired pneumonia in the elderly. Clin. Infect. Dis. Off. Publ. Infect. Dis. Soc. Am..

[29] Dias S.P., Brouwer M.C., van de Beek D. (2022). Sex and Gender Differences in Bacterial Infections. Infect. Immun..

[30] Ueshima J., Momosaki R., Shimizu A., Motokawa K., Sonoi M., Shirai Y., Uno C., Kokura Y., Shimizu M., Nishiyama A. (2021). Nutritional Assessment in Adult Patients with Dysphagia: A Scoping Review. Nutrients.

[31] Raad S., Smith C., Allen K. (2019). Nutrition Status and Chronic Obstructive Pulmonary Disease: Can We Move Beyond the Body Mass Index?. Nutr. Clin. Pract..

[32] Komiya K., Ishii H., Umeki K., Mizunoe S., Okada F., Johkoh T., Kadota J.-I. (2013). Impact of aspiration pneumonia in patients with community-acquired pneumonia and healthcare-associated pneumonia: A multicenter retrospective cohort study. Respirology.

[33] Whitlock G., Lewington S., Sherliker P., Clarke R., Emberson J., Halsey J., Qizilbash N., Collins R., Peto R., Prospective Studies Collaboration (2009). Body-mass index and cause-specific mortality in 900 000 adults: Collaborative analyses of 57 prospective studies. Lancet.

[34] Schembri S., Anderson W., Morant S., Winter J., Thompson P., Pettitt D., MacDonald T.M., Winter J.H. (2009). A predictive model of hospitalisation and death from chronic obstructive pulmonary disease. Respir. Med..

[35] Roche M., Rondeau P., Singh N.R., Tarnus E., Bourdon E. (2008). The antioxidant properties of serum albumin. FEBS Lett..

[36] Anraku M., Chuang V.T., Maruyama T., Otagiri M. (2013). Redox properties of serum albumin. Biochim. Biophys. Acta.

[37] Bak S.H., Kwon S.O., Han S.S., Kim W.J. (2019). Computed tomography-derived area and density of pectoralis muscle associated disease severity and longitudinal changes in chronic obstructive pulmonary disease: A case control study. Respir. Res..

[38] Sheth J.S., Xia M., Murray S., Martinez C.H., Meldrum C.A., Belloli E.A., Salisbury M.L., White E.S., Holtze C.H., Flaherty K.R. (2019). Frailty and geriatric conditions in older patients with idiopathic pulmonary fibrosis. Respir. Med..

[39] Yoshikawa H., Komiya K., Yamamoto T., Fujita N., Oka H., Okabe E., Yamasue M., Umeki K., Rubin B.K., Hiramatsu K. (2021). Quantitative assessment of erector spinae muscles and prognosis in elderly patients with pneumonia. Sci. Rep..

[40] O’Connell D.G., Brewer J.F., Man T.H., Weldon J.S., Hinman M.R. (2016). The Effects of Forced Exhalation and Inhalation, Grunting, and Valsalva Maneuver on Forehand Force in Collegiate Tennis Players. J. Strength Cond. Res..

[41] Tanimura K., Sato S., Fuseya Y., Hasegawa K., Uemasu K., Sato A., Oguma T., Hirai T., Mishima M., Muro S. (2016). Quantitative Assessment of Erector Spinae Muscles in Patients with Chronic Obstructive Pulmonary Disease. Novel Chest Computed Tomography-derived Index for Prognosis. Ann. Am. Thorac. Soc..

[42] Cederholm T., Jensen G.L., Correia M., Gonzalez M.C., Fukushima R., Higashiguchi T., Baptista G., Barazzoni R., Blaauw R., Coats A.J.S. (2019). GLIM criteria for the diagnosis of malnutrition—A consensus report from the global clinical nutrition community. J. Cachexia Sarcopenia Muscle.

[43] Negewo N.A., Gibson P.G., McDonald V.M. (2015). COPD and its comorbidities: Impact, measurement and mechanisms. Respirology.

[44] Caminati A., Lonati C., Cassandro R., Elia D., Pelosi G., Torre O., Zompatori M., Uslenghi E., Harari S. (2019). Comorbidities in idiopathic pulmonary fibrosis: An underestimated issue. Eur. Respir. Rev..

[45] Reyes S., Morel M.D.P., Kostka J., Nicolau D.P. (2021). Duration of antibiotic therapy for Enterobacterales and Pseudomonas aeruginosa: A review of recent evidence. Curr. Opin. Infect. Dis..

[46] Kosmidis C., Denning D.W. (2015). The clinical spectrum of pulmonary aspergillosis. Thorax.

[47] Bongomin F., Asio L.G., Baluku J.B., Kwizera R., Denning D.W. (2020). Chronic Pulmonary Aspergillosis: Notes for a Clinician in a Resource-Limited Setting Where There Is No Mycologist. J. Fungi.

[48] Gubbins P.O., Heldenbrand S. (2010). Clinically relevant drug interactions of current antifungal agents. Mycoses.

[49] Kuroishi S., Nakamura Y., Hayakawa H., Shirai M., Nakano Y., Yasuda K., Suda T., Nakamura H., Chida K. (2008). Mycobacterium avium complex disease: Prognostic implication of high-resolution computed tomography findings. Eur. Respir. J..

[50] Palaci M., Dietze R., Hadad D.J., Ribeiro F.K.C., Peres R.L., Vinhas S.A., Maciel E.L.N., Dettoni V.D.V., Horter L., Boom W.H. (2007). Cavitary disease and quantitative sputum bacillary load in cases of pulmonary tuberculosis. J. Clin. Microbiol..

[51] Page M.J., McKenzie J.E., Bossuyt P.M., Boutron I., Hoffmann T.C., Mulrow C.D., Shamseer L., Tetzlaff J.M., Akl E.A., Brennan S.E. (2021). The PRISMA 2020 statement: An updated guideline for reporting systematic reviews. BMJ.

